# Supplementary material for: Mixed tin-lead perovskites with balanced crystallization and oxidation barrier for all-perovskite tandem solar cells
Source: Nat Commun. 2024 Mar 14;15:2324. doi: 10.1038/s41467-024-46679-w (PMC10940575; doi:10.1038/s41467-024-46679-w)
Supplement: Supplementary file 1 — Supplementary Information [file 41467_2024_46679_MOESM1_ESM.pdf]

Supplementary Information for:

Mixed tin-lead perovskites with balanced crystallization and oxidation barrier for all-perovskite tandem solar cells

Jin Zhou[1], Shiqiang Fu[1], Shun Zhou[1], Lishuai Huang[1], Cheng Wang[1], Hongling Guan[1], Dexin Pu[1], Hongsen Cui[1], Chen Wang[1], Ti Wang[1], Weiwei Meng[2]✉, Guojia Fang[1]✉, Weijun Ke[1]✉

[1]Key Laboratory of Artificial Micro- and Nano-structures of Ministry of Education of China, School of Physics and Technology, Wuhan University, Wuhan, China.

[2]South China Academy of Advanced Optoelectronics, South China Normal University, Guangzhou, China.

✉e-mail: wwmeng@m.scnu.edu.cn; gjfang@whu.edu.cn; weijun.ke@whu.edu.cn

Table of Contents

Supplementary Figures 1-22

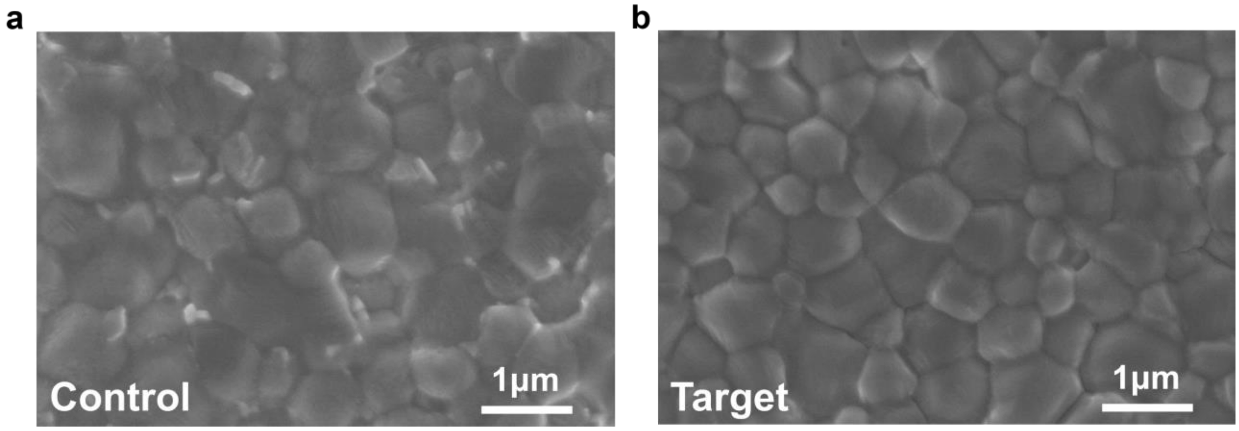

Supplementary Figure 1. Top-view SEM images of mixed Sn-Pb perovskite films (a) without and (b) with the doping of CPGCl.

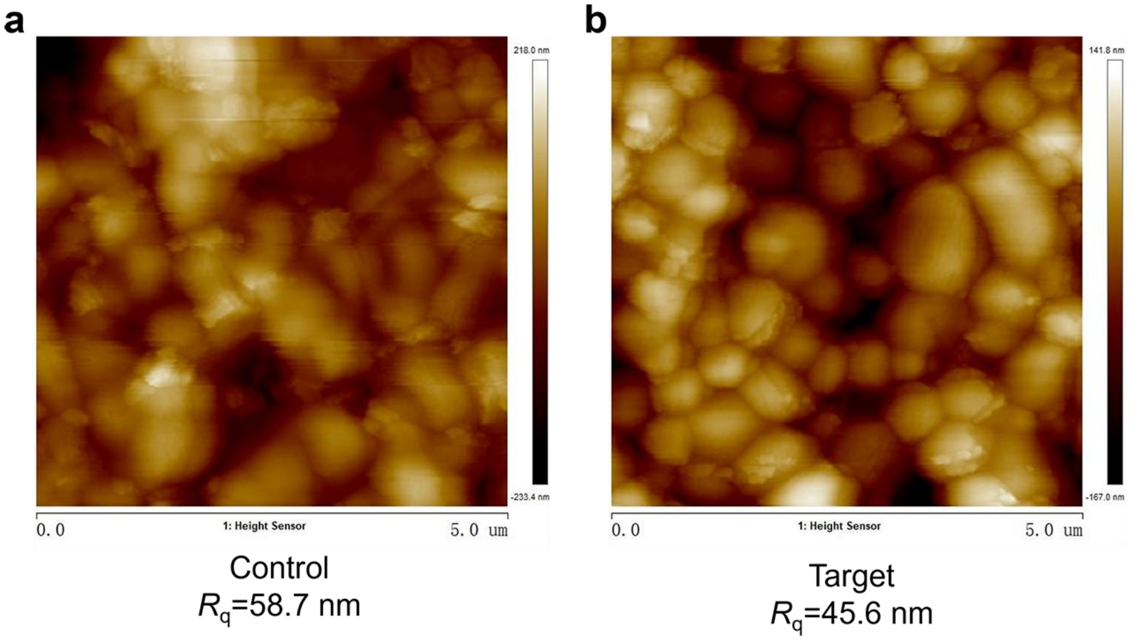

Supplementary Figure 2. AFM images of (a) control and (b) target Sn-Pb perovskite films.

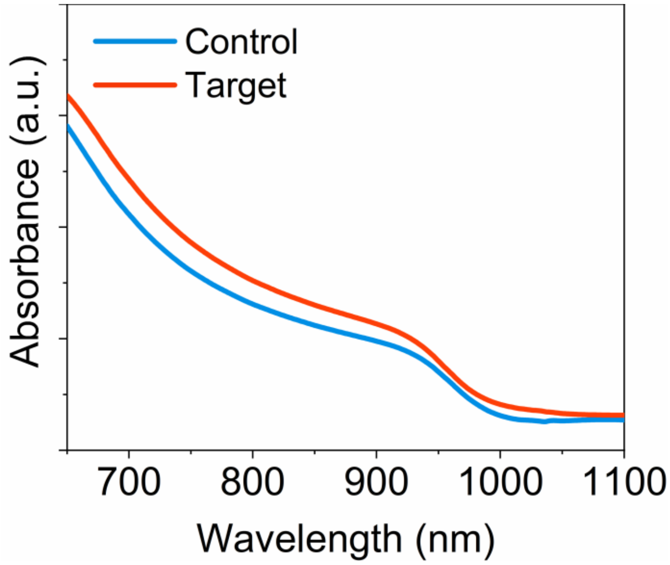

Supplementary Figure 3. UV-Vis-NIR absorption spectra of control and CPGCl-modified perovskite films.

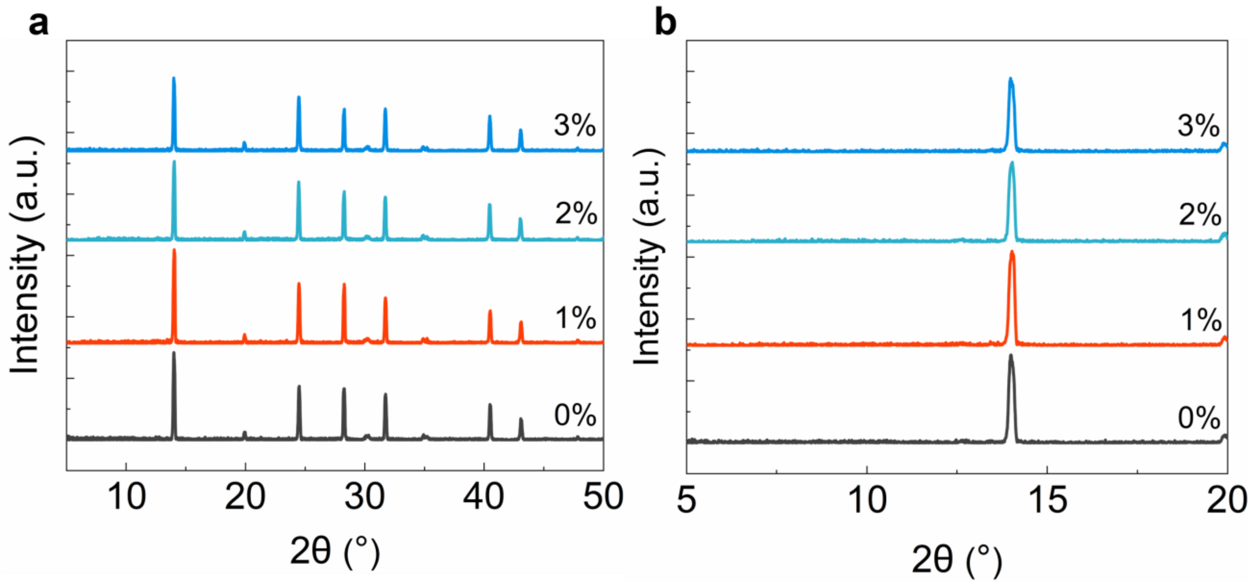

Supplementary Figure 4. XRD patterns, with different 2θ ranges of (a) 5-50° and (b) 5-20°, of perovskite films doped with various concentrations of CPGCl.

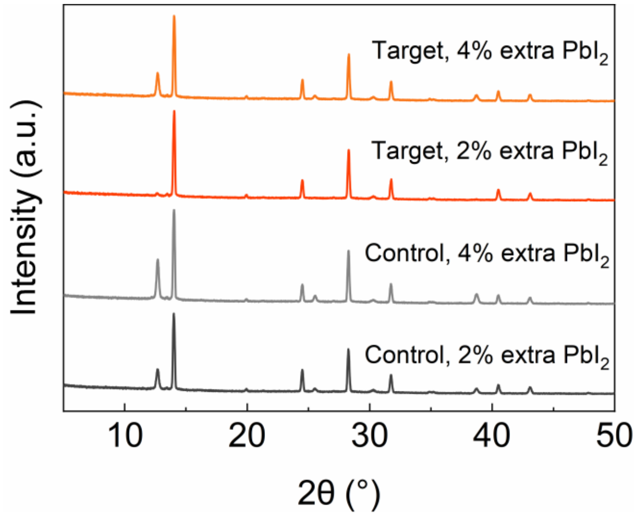

Supplementary Figure 5. XRD patterns of control and target Sn-Pb perovskite films with overdoses of PbI_2 (2% and 4%).

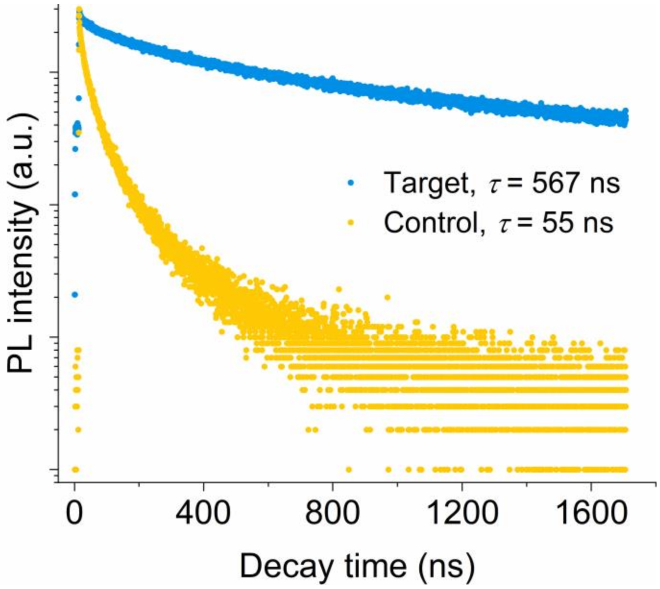

Supplementary Figure 6. TRPL spectra of a control film and a target CPGCl-modified perovskite film. The average carrier lifetime τ was calculated by $\tau = \frac{A_1\tau_1^2+A_2\tau_2^2}{A_1\tau_1+A_2\tau_2}$, where τ_1 and τ_2 denote the decay time constants, and A_1 and A_2 are the corresponding amplitudes of the decay, respectively. The samples were fabricated by spin-coating the perovskite precursors directly onto the glass substrates.

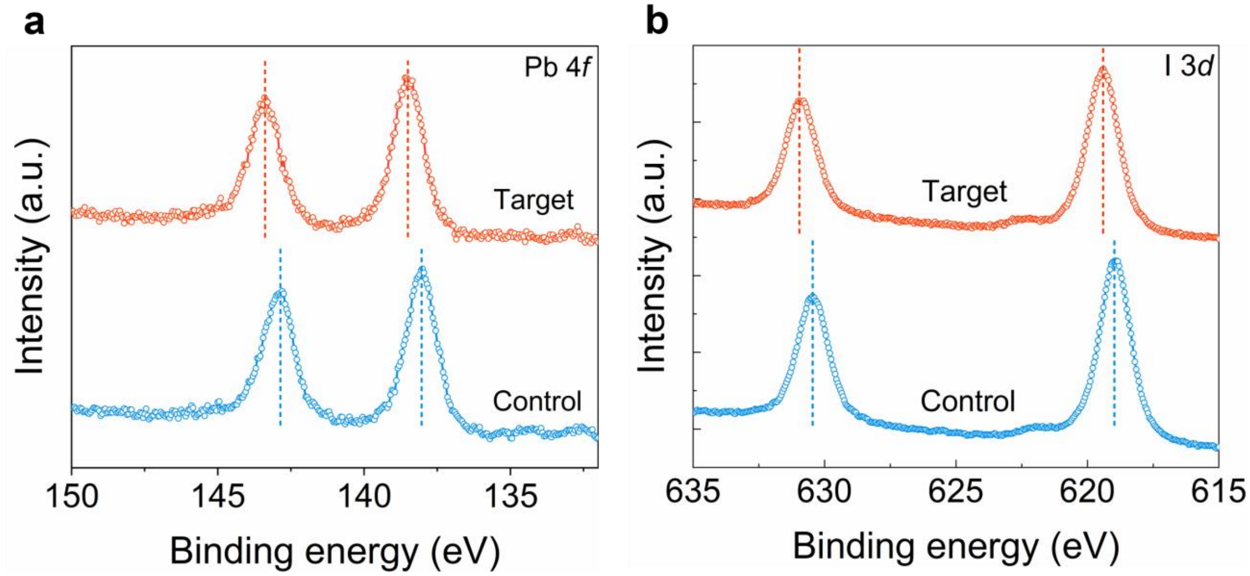

Supplementary Figure 7. XPS spectra of (a) Pb 4*f* and (b) I 3*d* core energy levels correlated to control and target Sn-Pb perovskite films.

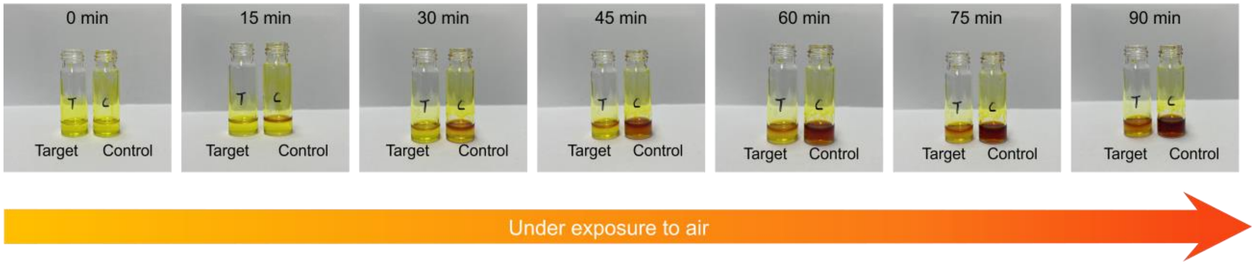

Supplementary Figure 8. Photographs of control and target Sn-Pb precursor solutions exposed to ambient air for varying durations.

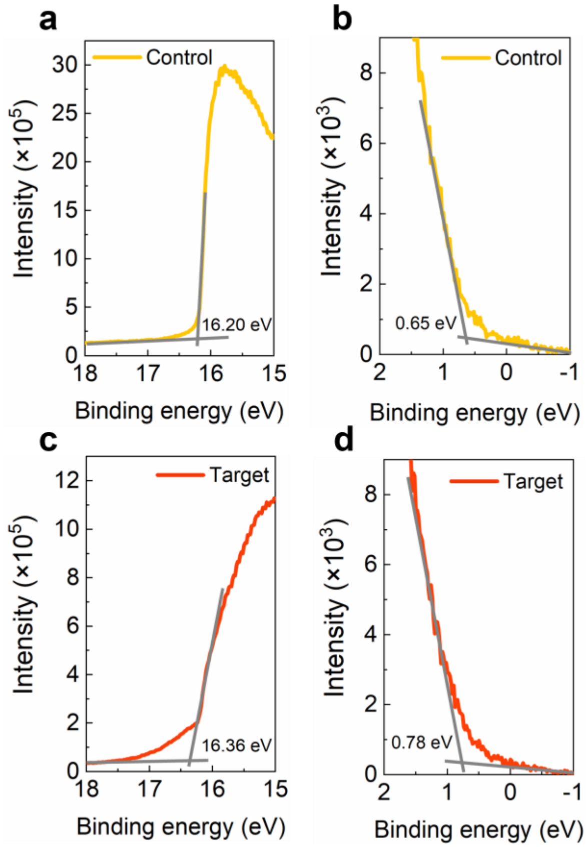

Supplementary Figure 9. UPS spectra of (a, b) control and (c, d) target Sn-Pb perovskites.

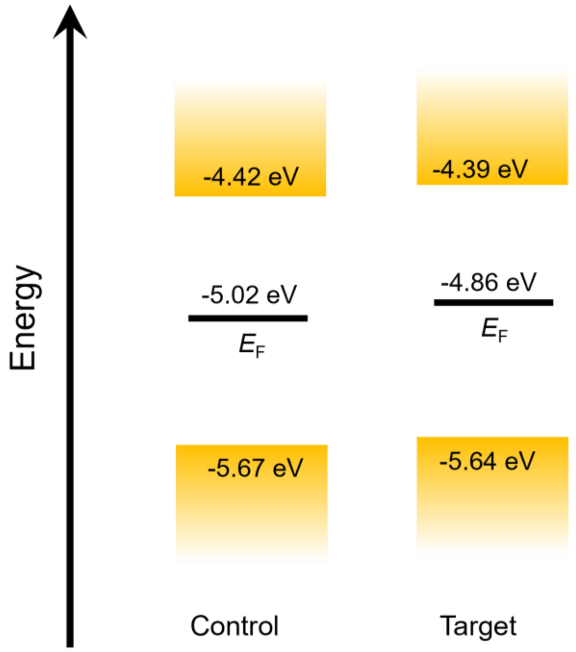

Supplementary Figure 10. The energy level alignment of control and target perovskites. Fermi level is denoted by E_F.

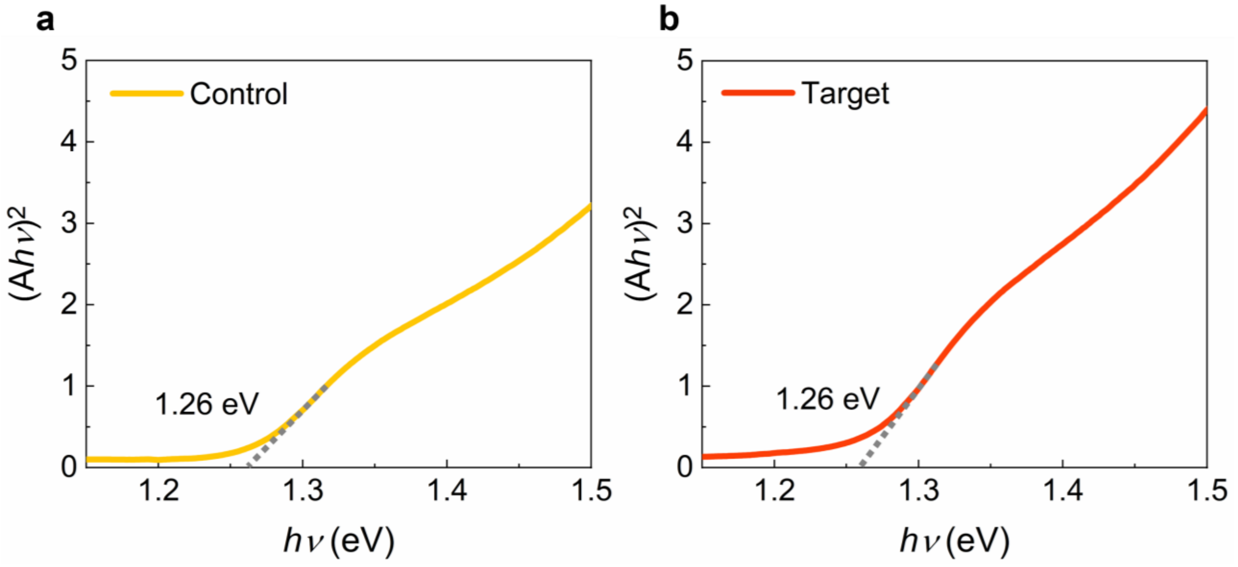

Supplementary Figure 11. Tauc plots of the perovskite films (a) without and (b) with CPGCl doping.

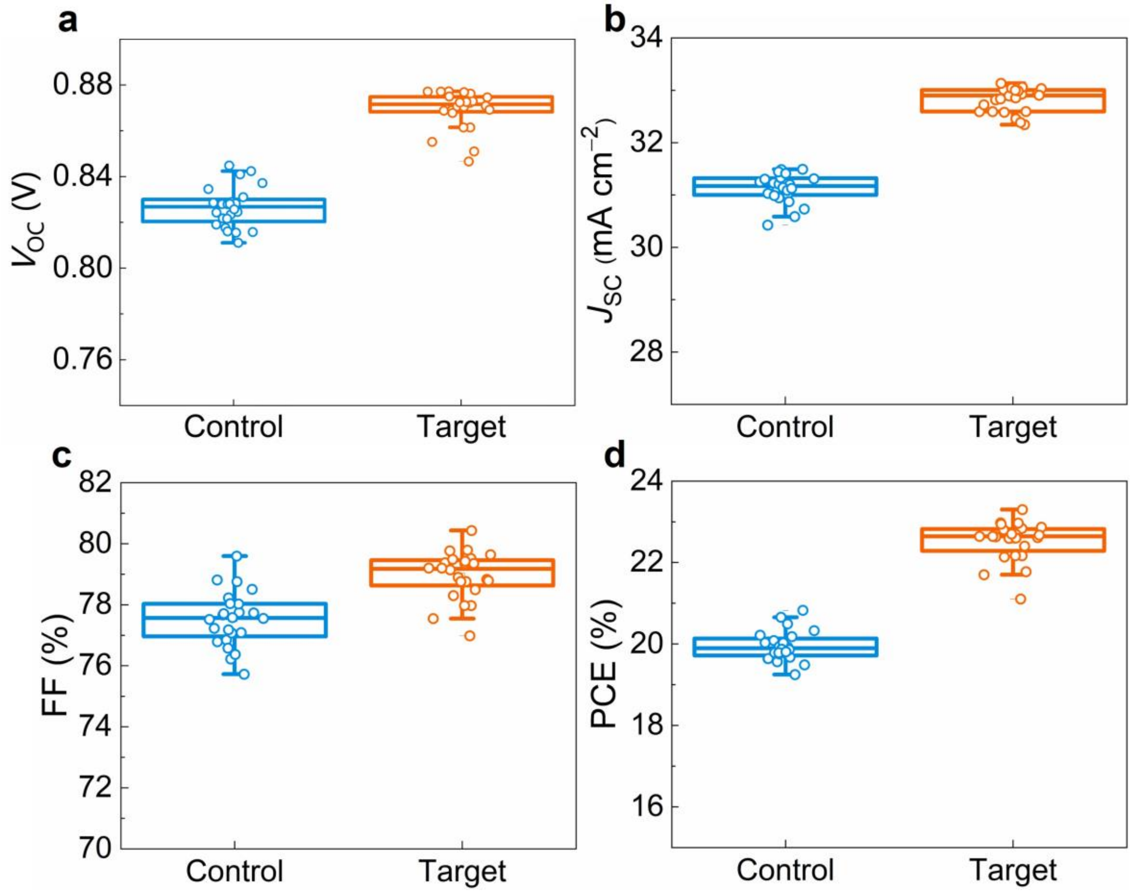

Supplementary Figure 12. Box plots showing the statistical distribution of (a) V_{OC}; (b) J_{SC}; (c) FF; and (d) PCE of the control and target single-junction mixed Sn-Pb PSCs (24 devices for each type).

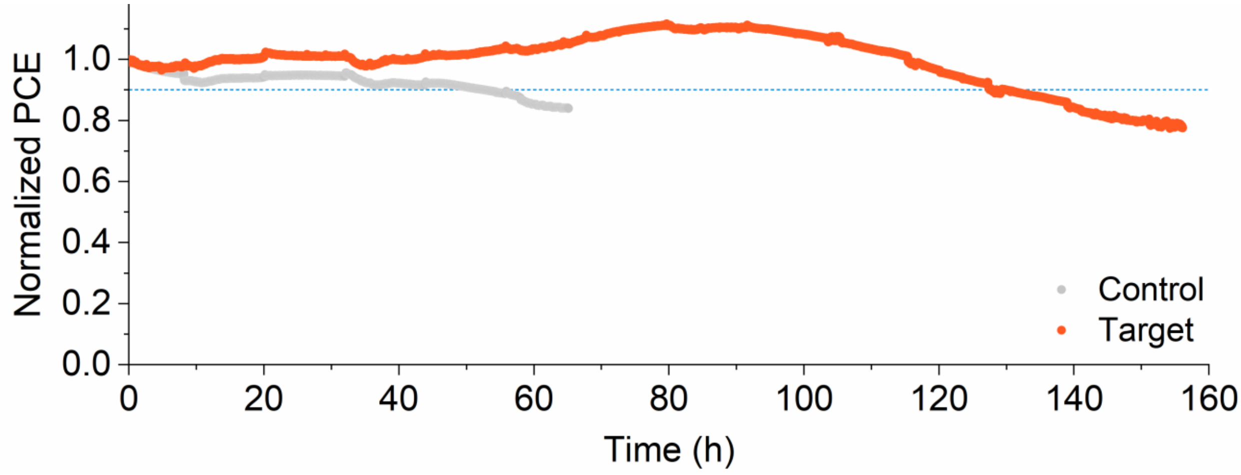

Supplementary Figure 13. Stability tests of unencapsulated control and target Sn-Pb PSCs under MPP tracking in an N_2-filled glovebox at approximately 55 °C. The slight fluctuations observed can be primarily attributed to the MPP tracking software and minor environmental variations.

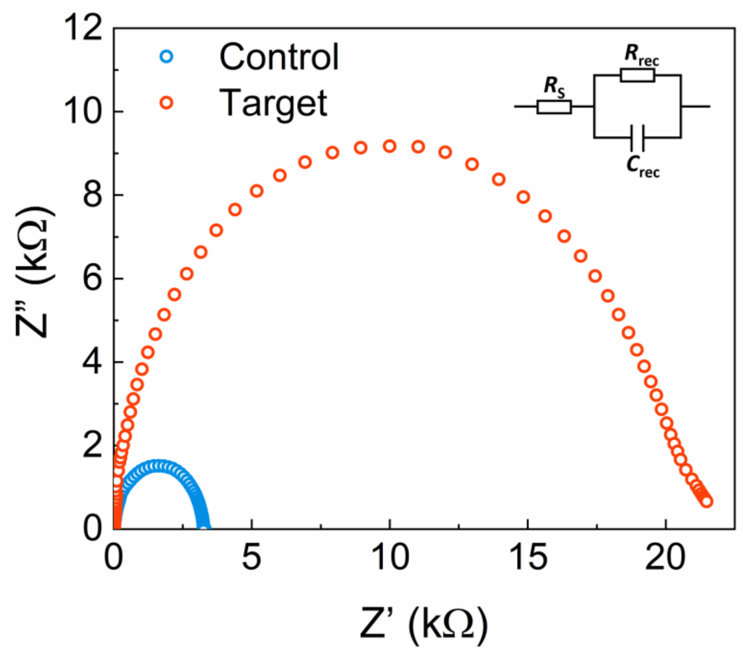

Supplementary Figure 14. Electronic impedance spectroscopy (EIS) plots of control and target Sn-Pb PSCs. The insect shows the equivalent circuit diagram of EIS measurements.

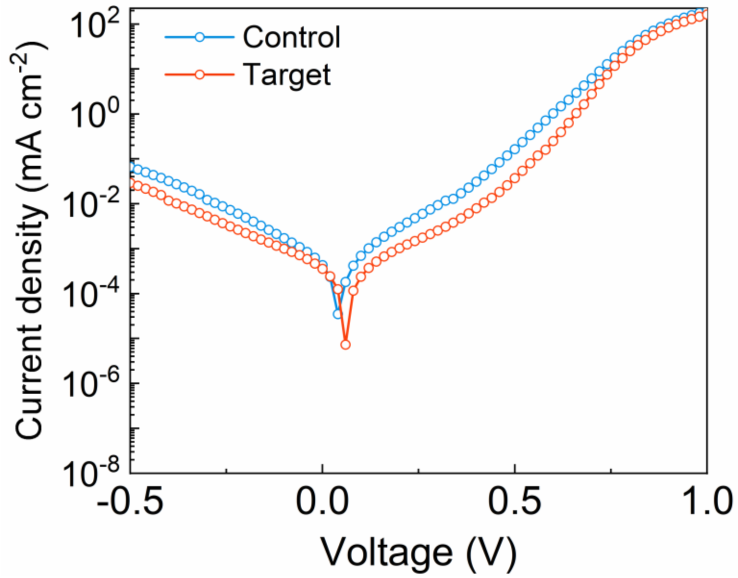

Supplementary Figure 15. Dark *J-V* curves of representative control and target CPGCl-treated Sn-Pb PSCs.

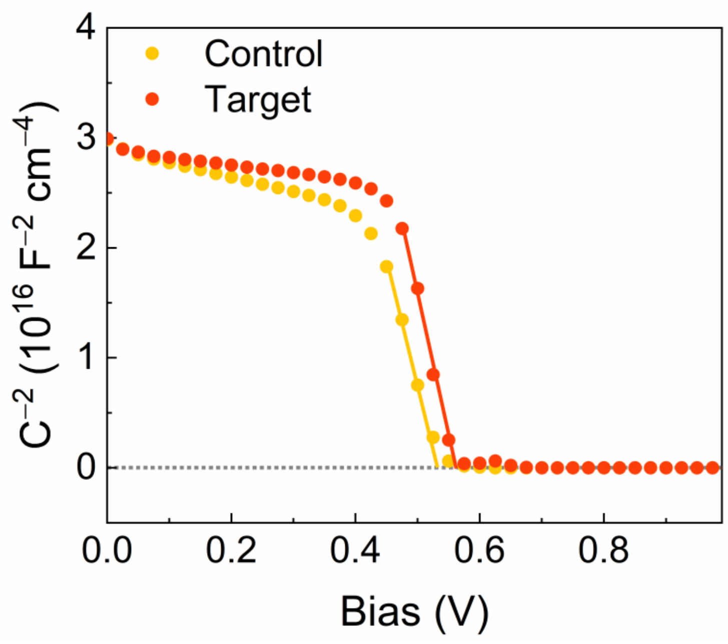

Supplementary Figure 16. Mott-Schottky plots of control and target CPGCl-treated Sn-Pb PSCs.

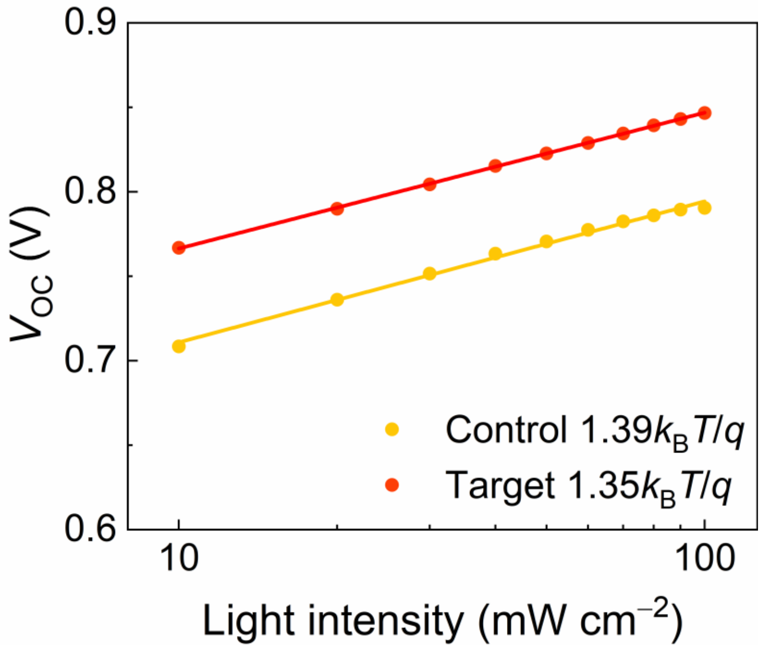

Supplementary Figure 17. Dependence of V_{OC} on light intensity for control and target CPGCl-treated Sn-Pb PSCs.

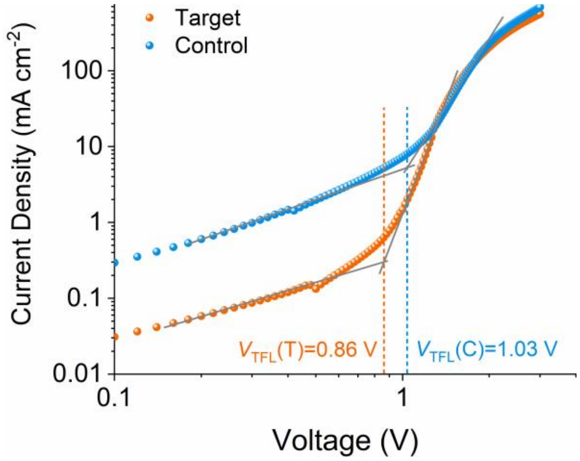

Supplementary Figure 18. SCLC curves of control and target hole-only devices. The configuration of samples was ITO/PEDOT: PSS/perovskite/gallium(III) acetylacetonate@poly (3-hexylthiophene) (mass ratio: 1:10)/Au.

中国认可
国际互认
检测
TESTING
CNAS L8490

Test and Calibration Center of New Energy Device and Module,
Shanghai Institute of Microsystem and Information Technology,
Chinese Academy of Sciences (SIMIT)

Measurement Report

Report No. 23TR041902

**Client Name**	Wuhan University
**Client Address**	299 Bayi Road, Wuchang District, Wuhan, Hubei Province
**Sample**	Perovskite/Perovskite Tandem Photovoltaic Cell
**Manufacturer**	Wuhan University
**Measurement Date**	19th April, 2023

**Performed by:**	Qiang Shi Qiang Shi	**Date:** 19/04/2023
**Reviewed by:**	Wenjie Zhao Wenjie Zhao	**Date:** 19/04/2023
**Approved by:**	Zhengxin Liu Zhengxin Liu	**Date:** 22/05/2023

Address: No.235 Chengbei Road, Jiading, Shanghai **Post Code:**201800
E-mail: solarcell@mail.sim.ac.cn **Tel:** +86-021-69976921

The measurement report without signature and seal are not valid.
This report shall not be reproduced, except in full, without the approval of SIMIT.

Sample Information

Sample Type	Perovskite/Perovskite Tandem photovoltaic cell
Serial No.	4-3#
Lab Internal No.	23041901-1#
Measurement Item	I-V characteristic
Measurement Environment	24.2±2.0°C, 44.1±5.0%R.H

Measurement of I-V characteristic

**Reference cell**	PVM1121
**Reference cell Type**	mono-Si, WPVS, calibrated by NREL (Certificate No. ISO 2075)
**Calibration Value/Date of Calibration for Reference cell**	144.53mA/ Feb. 2023
**Measurement Conditions**	Standard Test Condition (STC):<br>Spectral Distribution: AM1.5 according to IEC 60904-3 Ed.3,<br>Irradiance: 1000±50W/m^2, Temperature: 25±2°C
**Measurement Equipment/ Date of Calibration**	AAA Steady State Solar Simulator (YSS-T155-2M) / July.2022<br>IV test system (ADCMT 6246) / June. 2022<br>SR Measurement system (CEP-25ML-CAS) / April.2022<br>Measuring Microscope (MF-B2017C) / July.2022
**Measurement Method**	I-V Measurement:<br>Logarithmic sweep in both directions (Voc to Isc and Isc to Voc) during one flash based on IEC 60904-1:2006;<br>Spectral Mismatch factor was calculated according to IEC 60904-7 and I-V correction according to IEC 60891.
**Measurement Uncertainty**	Area: 1.0%(k=2); Isc: 2.1%(k=2); Voc: 1.0%(k=2);<br>Pmax: 2.7%(k=2); Eff: 2.8%(k=2)

====Measurement Results ====

	Forward Scan (Isc to Voc)		Reverse Scan (Voc to Isc)	
Area		6.94 mm^2		
Isc	1.138	mA	1.129	mA
Voc	2.124	V	2.130	V
Pmax	1.844	mW	1.898	mW
Ipm	1.002	mA	1.026	mA
Vpm	1.840	V	1.851	V
FF	76.31	%	78.94	%
Eff	26.57	%	27.35	%

- Spectral Mismatch Factor: SMM_{top}=1.0068, SMM_{bot}=0.9942.
- Designated illumination area defined by a thin metal mask was measured by a measuring microscope.
- Test results listed in this measurement report refer exclusively to the mentioned test sample.
- The results apply only at the time of the test, and do not imply future performance.

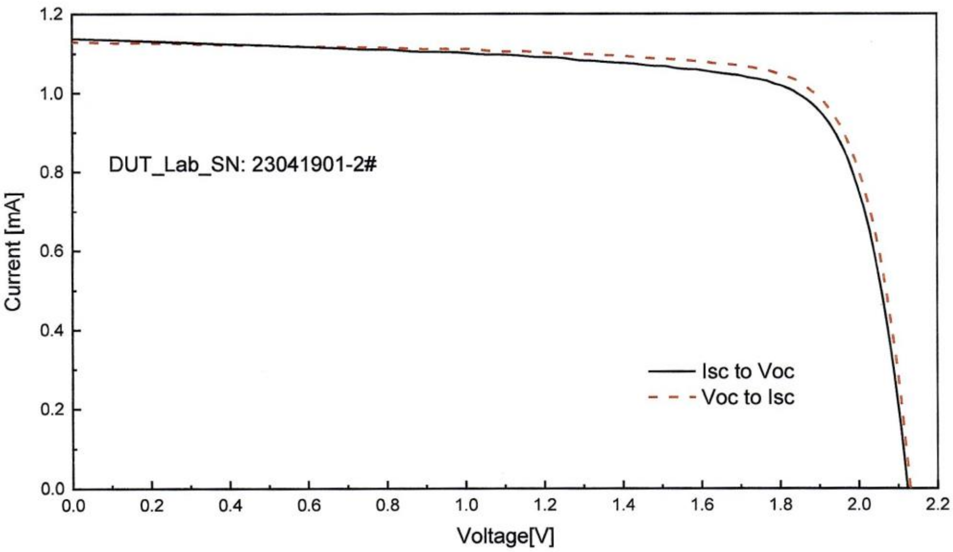

Fig.1 I-V curves of the measured sample

Report No. 23TR041902

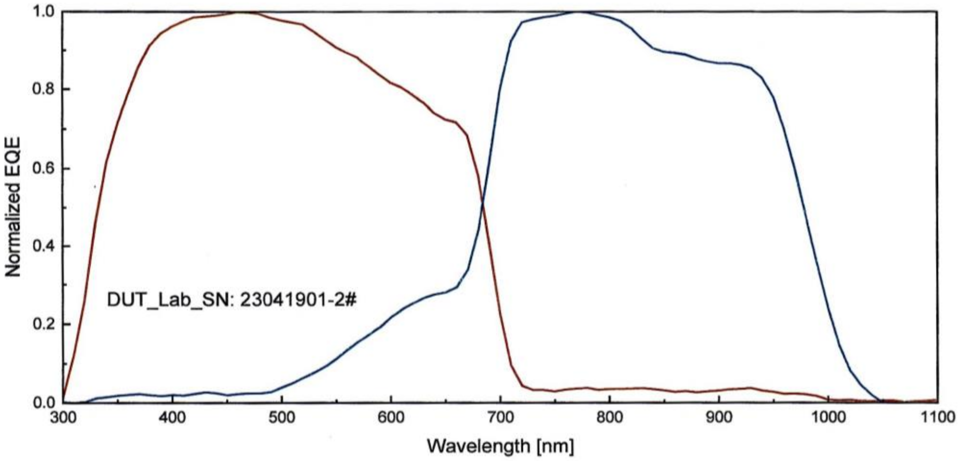

Fig.2 Normalized EQE curve of the measured sample

-------End of Report-------

4 / 4

Supplementary Figure 19. Certification report for a representative all-perovskite tandem cell based on a CPGCl-modified Sn-Pb subcell by Shanghai Institute of Microsystem and Information Technology (*SIMIT*).

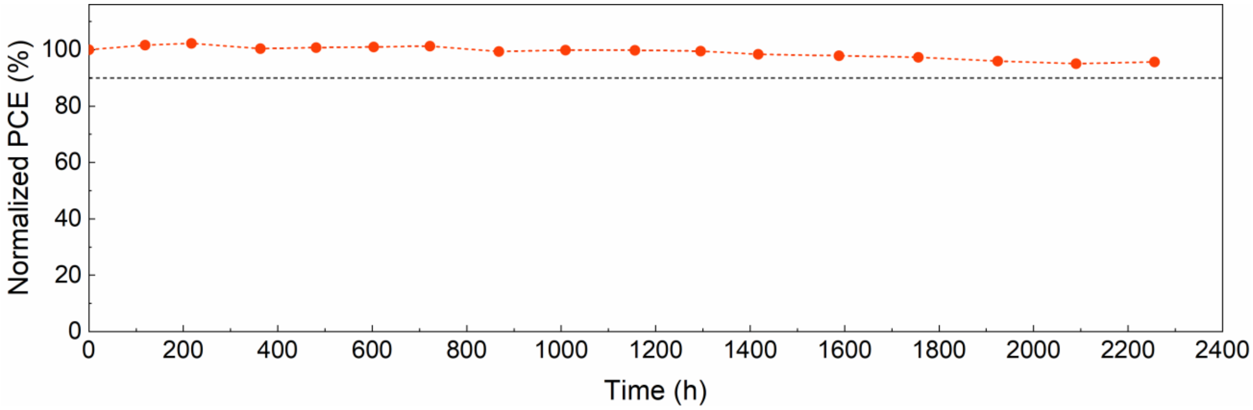

Supplementary Figure 20. Shelf-storage stability of a representative unencapsulated all-perovskite tandem solar cell. The device was stored in an N_2-filled glovebox in the dark.

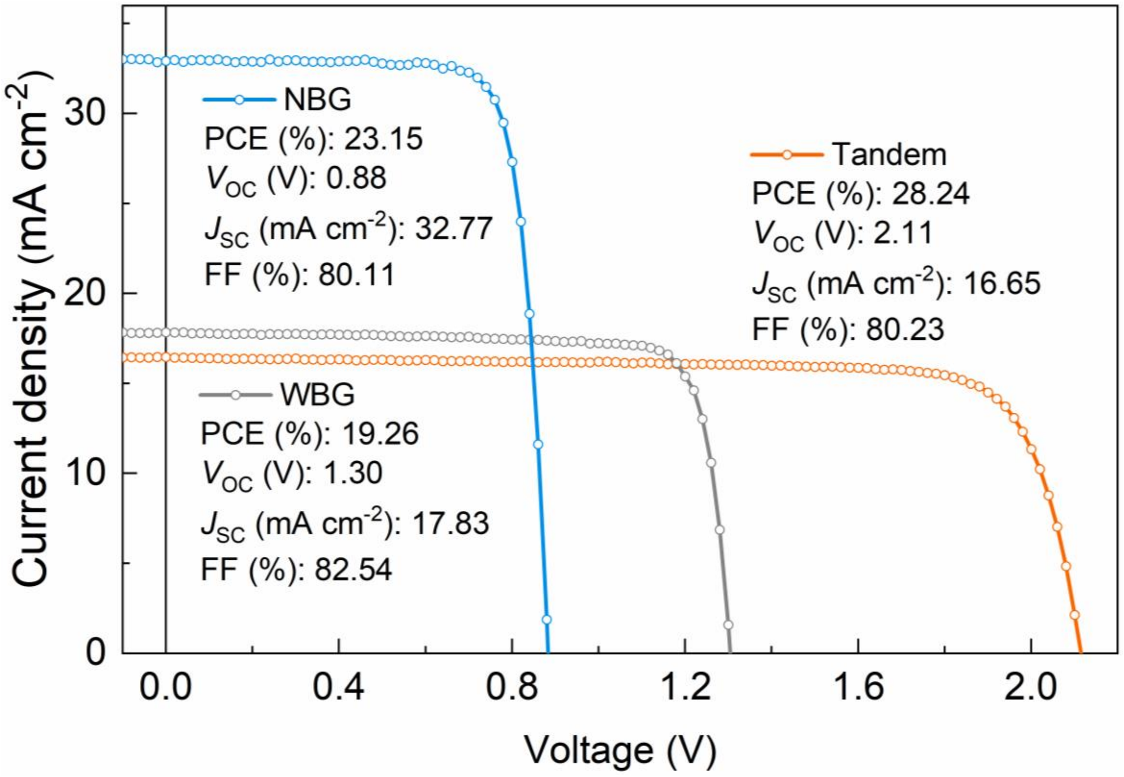

Supplementary Figure 21. *J-V* curves of the best-performing NBG, WBG, and 2T all-perovskite tandem solar cells.

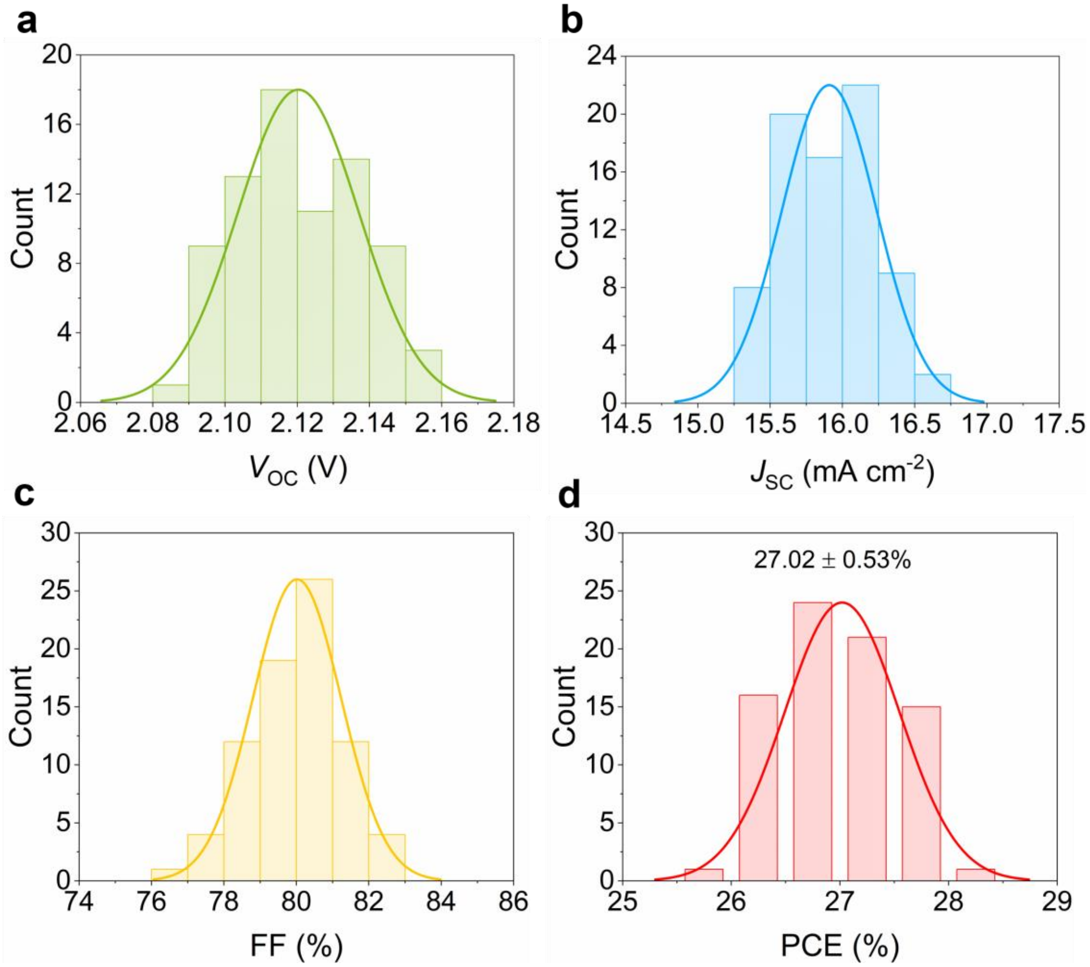

Supplementary Figure 22. Photovoltaic performance of 2T all-perovskite tandem cells (78 devices): (a) V_{OC}; (b) J_{SC}; (c) FF; (d) PCE.
