# Supplementary material for: Mixed tin-lead perovskites with balanced crystallization and oxidation barrier for all-perovskite tandem solar cells
Source: Nat Commun. 2024 Mar 14;15:2324. doi: 10.1038/s41467-024-46679-w (PMC10940575; doi:10.1038/s41467-024-46679-w)
Supplement: Supplementary file 3 — Reporting Summary [file 41467_2024_46679_MOESM3_ESM.pdf]

nature portfolio

Corresponding author(s): Weiwei Meng, Guojia Fang, and Weijun Ke

Solar Cells Reporting Summary

Nature Portfolio wishes to improve the reproducibility of the work that we publish. This form is intended for publication with all accepted papers reporting the characterization of photovoltaic devices and provides structure for consistency and transparency in reporting. Some list items might not apply to an individual manuscript, but all fields must be completed for clarity.

For further information on Nature Research policies, including our data availability policy, see Authors & Referees.

Experimental design

Please check the following details are reported in the manuscript, and provide a brief description or explanation where applicable.

1. Dimensions

Area of the tested solar cells

- [x] Yes — Methods Section, Film and device characterization
- [ ] No — *Explain why this information is not reported/not relevant.*

Method used to determine the device area

- [x] Yes — Methods Section, Film and device characterization
- [ ] No — *Explain why this information is not reported/not relevant.*

2. Current-voltage characterization

Current density-voltage (J-V) plots in both forward and backward direction

- [x] Yes — Figure 3d, Figure 4c, and Table 1
- [ ] No

Voltage scan conditions

- [x] Yes — Methods Section, Film and device characterization.
- [ ] No — *Explain why this information is not reported/not relevant.*

Test environment

- [x] Yes — In an N2-filled glove box at room temperature.
- [ ] No — *Explain why this information is not reported/not relevant.*

Protocol for preconditioning of the device before its characterization

- [ ] Yes — *Provide a description of the protocol.*
- [x] No — No preconditioning is required for our cell characterization.

Stability of the J-V characteristic

- [x] Yes — Figure 3e, Figure 4d. The stabilized power output efficiencies near the maximum power point voltages were provided.
- [ ] No — *Explain why this information is not reported/not relevant.*

3. Hysteresis or any other unusual behaviour

Description of the unusual behaviour observed during the characterization

- [x] Yes — Negligible hysteresis was found.
- [ ] No — *Explain why this information is not reported/not relevant.*

Related experimental data

- [x] Yes — J-V curves under reverse and forward scans were provided.
- [ ] No — *Explain why this information is not reported/not relevant.*

4. Efficiency

External quantum efficiency (EQE) or incident photons to current efficiency (IPCE)

- [x] Yes — EQE curves were provided in Figure 3f and Figure 4e.
- [ ] No — *Explain why this information is not reported/not relevant.*

A comparison between the integrated response under the standard reference spectrum and the response measure under the simulator

- [x] Yes — The integrated Jsc values obtained from EQE agree well with the Jsc determined from the J–V measurements.
- [ ] No — *Explain why this information is not reported/not relevant.*

For tandem solar cells, the bias illumination and bias voltage used for each subcell

☒ Yes
☐ No

Methods Section, Film and device characterization

Explain why this information is not reported/not relevant.

5. Calibration

Light source and reference cell or sensor used for the characterization

☒ Yes
☐ No

Methods Section, Film and device characterization

Explain why this information is not reported/not relevant.

Confirmation that the reference cell was calibrated and certified

☒ Yes
☐ No

The light intensity was set at 100 mW cm−2 and calibrated with a certified WPVS standard solar reference cell (SRC-2020, Enlitech; traceable to NREL) before measurements.

Explain why this information is not reported/not relevant.

Calculation of spectral mismatch between the reference cell and the devices under test

☐ Yes
☒ No

Provide a value of the spectral mismatch and/or a description of how it has been taken into account in the measurements.

The light spectrum used for measurements matches well with the reference silicon cell, and we did not calculate the spectral mismatch between the reference cell and the tested devices.

6. Mask/aperture

Size of the mask/aperture used during testing

☒ Yes
☐ No

The area of the aperture mask was 0.070225 cm2.

Explain why this information is not reported/not relevant.

Variation of the measured short-circuit current density with the mask/aperture area

☐ Yes
☒ No

Report the difference in the short-circuit current density values measured with the mask and aperture area.

Negligible

7. Performance certification

Identity of the independent certification laboratory that confirmed the photovoltaic performance

☒ Yes
☐ No

A representative tandem solar cell was certified by Shanghai Institute of Measurement and Testing Technology (SIMT).

Explain why this information is not reported/not relevant.

A copy of any certificate(s)

☒ Yes
☐ No

Supplementary Figure 19

Explain why this information is not reported/not relevant.

8. Statistics

Number of solar cells tested

☒ Yes
☐ No

Provided in the manuscript.

Explain why this information is not reported/not relevant.

Statistical analysis of the device performance

☒ Yes
☐ No

Provided in the manuscript and Supplementary Information.

Explain why this information is not reported/not relevant.

9. Long-term stability analysis

Type of analysis, bias conditions and environmental conditions

☒ Yes
☐ No

Figure 3g, Supplementary Figure 20, and Methods Section.

Explain why this information is not reported/not relevant.
